# Auditory Cortex Contributes to Discrimination of Pure Tones

**DOI:** 10.1523/ENEURO.0340-19.2019

**Published:** 2019-10-15

**Authors:** Conor O’Sullivan, Aldis P. Weible, Michael Wehr

**Affiliations:** 1 Institute of Neuroscience; 2 Department of Biology; 3 Department of Psychology, University of Oregon, Eugene, Oregon 97403

**Keywords:** auditory cortex, frequency discrimination, optogenetics, perceptual decisions

## Abstract

The auditory cortex is topographically organized for sound frequency and contains highly selective frequency-tuned neurons, yet the role of auditory cortex in the perception of sound frequency remains unclear. Lesion studies have shown that auditory cortex is not essential for frequency discrimination of pure tones. However, transient pharmacological inactivation has been reported to impair frequency discrimination. This suggests the possibility that successful tone discrimination after recovery from lesion surgery could arise from long-term reorganization or plasticity of compensatory pathways. Here, we compared the effects of lesions and optogenetic suppression of auditory cortex on frequency discrimination in mice. We found that transient bilateral optogenetic suppression partially but significantly impaired discrimination performance. In contrast, bilateral electrolytic lesions of auditory cortex had no effect on performance of the identical task, even when tested only 4 h after lesion. This suggests that when auditory cortex is destroyed, an alternative pathway is almost immediately adequate for mediating frequency discrimination. Yet this alternative pathway is insufficient for task performance when auditory cortex is intact but has its activity suppressed. These results indicate a fundamental difference between the effects of brain lesions and optogenetic suppression, and suggest the existence of a rapid compensatory process possibly induced by injury.

## Significance Statement

The role of auditory cortex in the perception of elementary sound properties has remained unclear. Here, we show that although damage to auditory cortex has no effect on an animal’s ability to discriminate sound frequency, auditory cortex is nevertheless involved in frequency discrimination when it is not damaged. These results suggest that the ability to recover from brain damage requires mechanisms beyond the loss of neural activity in the damaged part of the brain.

## Introduction

Neurons in auditory cortex are well tuned for frequency and are organized into multiple tonotopic maps across the cortical surface. Is auditory cortex involved in the perception and discrimination of sound frequencies? For pure tones, across a wide range of species and behavioral paradigms, the consensus view has been that the answer appears to be no. Although there are conflicting results, lesion studies have generally shown that frequency discrimination of pure tones is not affected by ablation of auditory cortex, even after extensive lesions of all known auditory cortical fields (Meyer and Woolsey, 1952; Butler et al., 1957; Thompson, 1960; Goldberg and Neff, 1961; Sellick, 1983; Buser and Imbert, 1992; Ohl et al., 1999; Talwar et al., 2001; Rybalko et al., 2006; Porter et al., 2011; Gimenez et al., 2015). Discrimination of more spectrotemporally complex sounds such as frequency-modulated tones is impaired by lesions of auditory cortex, suggesting that auditory cortex is recruited when task demands require spectral or temporal integration (Ohl et al., 1999). The effects of transient inactivation on pure tone discrimination (for example, with local muscimol application) have been inconsistent, with some studies reporting no effect while others report complete impairment (Talwar et al., 2001; Gimenez et al., 2015). The fact that some transient inactivation studies observed complete impairment suggests that auditory cortex could potentially be involved in frequency discrimination, and that the effects of lesions could differ from those of transient inactivation because of cortical reorganization or some other long-term recovery or compensatory processes. Lesion studies typically include at least several days of recovery after surgery, which could allow time for cortical or subcortical plasticity to eventually allow alternative structures or pathways to mediate frequency discrimination.

More recently, optogenetic suppression experiments in other neural systems have shown that acute suppression can reveal involvement of a brain structure in specific tasks even when lesions of the same structure have no effect (Goshen et al., 2011; Kumar et al., 2013; Otchy et al., 2015; Hong et al., 2018). For example, remote contextual fear memories are unaffected by hippocampal lesions, but can be abolished by transient optogenetic hippocampal suppression (Goshen et al., 2011). This suggests that a brain structure could be critically involved in specific functions when it is intact and “online,” despite the existence of alternative pathways that are adequate for that function. Optogenetic studies such as these are thus providing new insights into redundancy and interactive processing in the brain, aspects which can be both evolutionarily adaptive and experimentally vexing. Such findings also prompt a re-evaluation of conclusions that structures (such as auditory cortex) are not involved in a task (such as frequency discrimination) based on lesions that produced no deficit in task performance.

Here, we compared the effects of lesions and optogenetic suppression of auditory cortex on frequency discrimination in mice. For suppression, we used mice that expressed Channelrhodopsin2 in parvalbumin-expressing interneurons (PV-ChR2), and trained them in an operant task to discriminate the frequency of brief pure tones for a water reward. We found that transient bilateral optogenetic suppression partially but significantly impaired discrimination performance. In contrast, bilateral electrolytic lesions of auditory cortex had no effect on performance of the identical task, even when tested only 4 h after lesion. This suggests that when auditory cortex is destroyed, an alternative pathway is adequate for mediating frequency discrimination. Yet this alternative pathway is insufficient for task performance when auditory cortex is intact but has its activity suppressed. These results indicate a fundamental difference between the effects of brain lesions and optogenetic suppression, and suggest the existence of a rapid compensatory process possibly induced by injury.

## Materials and Methods

### Mice

For optogenetic suppression of auditory cortex, we used mice that were offspring from a cross of homozygous Pvalb-IRES-Cre (PV; 008069; The Jackson Laboratory) and homozygous CAG-ChR2-eYFP (ChR2; 012569; The Jackson Laboratory) lines, which are on a C57Bl6/J background (*n* = 19 mice in total). In these mice (PV-ChR2), ChR2 was expressed in PV-expressing (PV+) interneurons, with 97% specificity in auditory cortex (Moore and Wehr, 2013). We used C57Bl6/J mice that did not express ChR2 as controls (*n* = 5 mice; three were –/ChR2, 1 was –/–, 1 was GPR26-cre/–). For electrolytic lesion experiments, we used wild-type C57Bl6/J mice (*n* = 5 mice).

### Surgical procedures

We administered dexamethasone (0.1 mg/kg) and atropine (0.03 mg/kg) pre-surgically to reduce inflammation and respiratory irregularities. Surgical anesthesia was maintained with isoflurane (1.25–2.0%). For optogenetic manipulation, we implanted 200-μm optic fibers in each hemisphere at AP ∼2.3 mm (relative to bregma), ML 4.4 mm, and depth 0.5 mm below the dura (just dorsal to primary auditory cortex). The implants were painted with black acrylic paint to minimize light leakage. For electrophysiological verification of optogenetic suppression, we implanted two mice (not used in behavioral experiments) with a unilateral optrode array, consisting of eight tetrodes and a 200-μm fiber terminating 1 mm above the recording sites. The eight tetrodes passed through two 28-gauge stainless-steel hypodermic tubes, with four tetrodes per tube. The optic fiber was fixed in position immediately adjacent to, and between, these tubes. Tetrodes were made of 18-μm (25 μm coated) tungsten wire (California Fine Wire). The entire array was mounted on a custom microdrive. The optrode array was inserted vertically through a small craniotomy (2 × 1 mm) dorsal to auditory cortex, and cemented into place. For electrolytic lesions, we implanted a pair of stainless-steel wires (112 μm in diameter, 150 μm coated) spaced ∼750 μm apart into auditory cortex in each hemisphere. The Teflon coating was stripped 500 μm from the tips. Pairs were implanted at ML 4.2 mm, DV 1.0 mm, centered on AP –2.9 mm. We administered ketoprofen (4.0 mg/kg) postoperatively to minimize discomfort. Mice were housed individually following the surgery and were allowed 7 d of postoperative recovery.

### Histology

Brains of mice used for electrophysiological validation were sectioned (100 μm) in the coronal plane to verify the position of single neuron recording sites. Only data corresponding to tracks located within auditory cortex were included.

### Behavioral setup

Mice performed the task in sound-attenuating behavioral chambers. Within the chamber, mice were placed in a plastic arena, one wall of which contained three combination ports for lick-sensing and water delivery. Each port had an IR beam-break sensor, at which mice responded by licking, and a tube to deliver water rewards for correct responses. Sound stimuli were controlled by a computer running custom behavioral software (modified from Meier et al., 2011), and delivered through two speakers placed outside the arena facing the ports. Since laser illumination was delivered with blue light that could potentially be visible to the mouse, we used a color-matched blue strobe light (full-field illumination at ∼10 Hz) to mask laser stimulation. Mice were trained for 1 h each day for 5–7 d/week, corresponding to 300–500 trials and 1–2 g of water reward per day.

Before any behavioral training, the mice underwent surgical implantation of optical fibers or lesion electrodes (step 1; see Table 1). Mice started training with a simple lick-for-water task to familiarize them with the operation of the ports in the absence of sound stimuli (step 2, “free drinks”). Next, they advanced to the first stage of the main task (step 3). In the main task, mice requested trials by licking the center port, which triggered stimulus delivery. Mice responded by licking at the left port (for 4 kHz, 500-ms pure tones) or the right port (for 13 kHz, 500-ms pure tones). Correct responses triggered an 80 μl water reward followed by a 1-s delay before the next trial could be requested. Incorrect responses gave no water and produced an additional 1-s penalty timeout before the next trial. To increase the number of trials performed, some mice had their water rewards reduced to 40 or 60 μl. During an initial shaping stage of the main task (step 3), mice received water rewards at the center port for requesting trials (as well as for correct answers at the side ports) until reaching a rate of seven completed trials in 30 s. Once the mice achieved this rate of trials, the rewards for requesting at the center port were removed (step 4). In steps 3 and 4, we included “correction trials” to reduce the development of response bias to one side or the other. After an incorrect response, there was a 50% chance that a mouse would go into a correction trial sequence, in which the same stimulus was repeated until the mouse responded correctly. Correction trials provide contextual information that could conceivably allow a task strategy that did not depend solely on stimulus discrimination, so we disabled correction trials during the final training stage. After 400 trials at step 4, the penalty timeout for incorrect responses was increased to 3 s and correction trials were turned off (step 5). When mice were performing at 85% or higher on step 5 for ∼5 d, they were run for at least 2 d with fibers attached to the ferrules on their head but without light delivery, to allow the mice to become accustomed to the tethers. Then mice advanced to the final stage (step 6) for optogenetic suppression experiments, using one or more of the illumination protocols described below.

**Table 1.** Training steps, optogenetic suppression

Training step	Description	Advancement criterion
1. Surgery	Fiber implantation	1 d of water restriction after recovery
2. Free drinks	Ports give water, no stimulus	Trial rate (cannot trigger the same port repeatedly)
3. Requests rewarded	Rewards given for center-port trial requests and for correct responses	Trial rate (7 trials in 30 s)
4. Only correct responses rewarded	Rewards only given for correct responses	400 trials completed
5. Longer penalty	Increased timeout for incorrect responses, no correction trials	Sustained performance > ∼85%
6. Optogenetic suppression	Laser on for 10% of trials	N/A

Stimuli were 500-ms pure tones at two frequencies, 4 and 13 kHz. Sound levels were not identical for all behavior boxes (range: 73- to 82-dB SPL, mean: 77 dB, SD: 3 dB) but within a given box the sound levels were similar for the two frequencies (mean difference: 0.3 dB) and were consistent from day to day. Reaction times were measured from tone onset to response port entry, with video frame resolution (16.6 ms).

### Optogenetic suppression

To suppress auditory cortex, 445-nm wavelength (blue) diode laser pulses were delivered to the implanted bilateral optical fibers, with an output power of 9.5 mW as measured at the fiber tip (corresponding to an irradiance of 300 mW/mm^2^). In a previous study using identical fiber implantation and lasers, we electrophysiologically characterized the spatial extent of cortical suppression, which was 1750 μm at this power level (Weible et al., 2014). This extent includes all tonotopic fields of auditory cortex, throughout the cortical depth, but does not include thalamic, collicular, or other subcortical regions. In a subset of mice, we also tested a power level of 6.3 mW (200 mW/mm^2^), which has a spatial extent of 1500 μm. We used three different temporal patterns of light delivery, which we refer to as transient suppression, cycle suppression, and sustained suppression.

### Transient suppression

**Figure 1.**
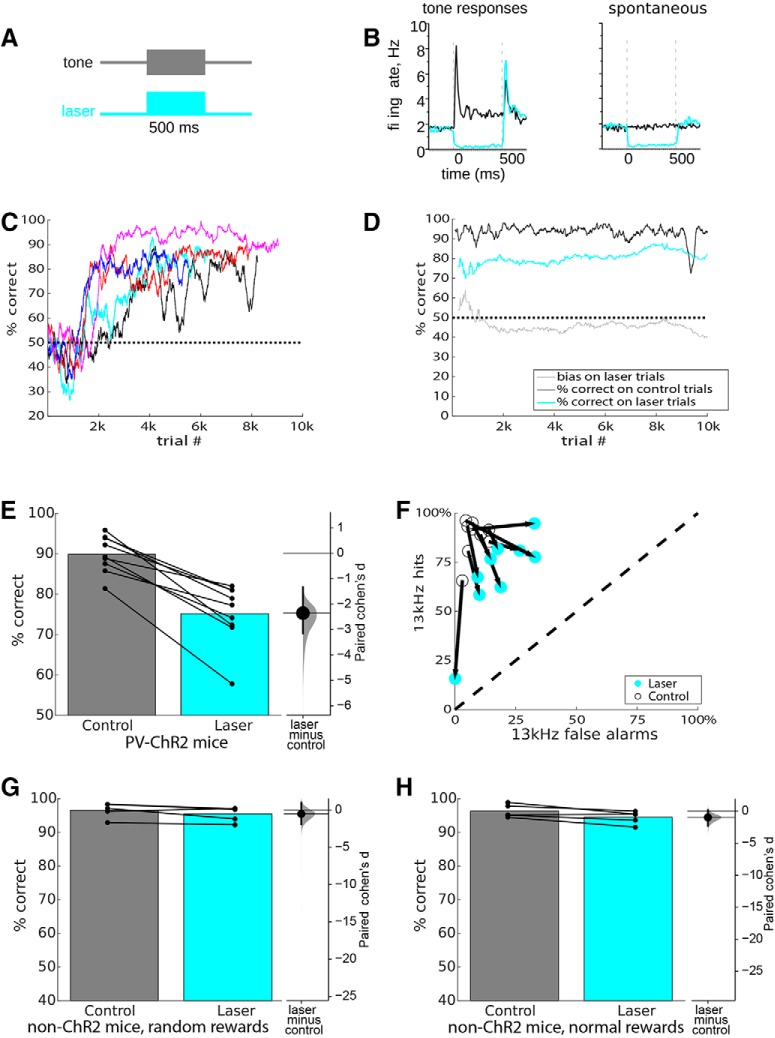
Transient optogenetic suppression of auditory cortex impaired pure tone discrimination. ***A***, Transient laser suppression used a 500-ms constant laser pulse at 300 mW/mm^2^ (9.5-mW total power) that completely overlapped with the 500-ms pure tone stimulus. Laser was delivered on a random 10% subset of trials. ***B***, Electrophysiological validation of optogenetic suppression in separate mice. Left, Responses to best-frequency tones averaged across 46 tone-responsive neurons and 50 repetitions under control conditions (black) and during a 500-ms constant laser pulse at 200 mW/mm^2^ (cyan). Right, Effect of illumination on spontaneous activity during silence, averaged across 90 neurons and 50 repetitions. Both tone responses and spontaneous activity were significantly suppressed. ***C***, Progression through training steps is shown for five example mice, aligned to the start of training. Performance was computed in a 200-trial sliding window. ***D***, The first 10,000 total trials in the suppression condition (step 6) for an example mouse (mouse rt.15) show a consistent effect of the laser, with minimal effects on bias. A 200-trial sliding window was used to separately compute accuracy over time on control trials (black) and laser trials (cyan). Since this would require ∼2000 total trials before having a sufficient sample to measure laser performance, we used a smaller window of 50 trials to measure performance for the initial 100 laser trials, and a 200-trial window for subsequent laser trials. We also measured the bias on laser trials (gray), with 50% corresponding to equal left and right responses. ***E***, Performance in overall percentage correct for each mouse for 10,000 total trials is shown by connected dots for control and laser trials. Bars represent mean performance for each trial type across all nine mice. Inset at right shows a Gardner–Altman estimation plot of effect size. The dot shows the paired Cohen’s *d*, the vertical bar shows the 95% confidence interval, and the gray shading shows the bootstrap sampling distribution of paired Cohen’s *d*. ***F***, To show stimulus-related effects, we used ROC analysis to separate each mouse’s performance into “hit rate” (percentage correct on 13-kHz trials) and “false alarm rate” (100 − percent correct on 4-kHz trials). The dashed line represents chance performance (50% correct), ranging from 100% leftward (4 kHz) responses at the lower left corner to 100% rightward (13 kHz) responses at the upper right corner. Performance for each mouse on control trials is represented with a large open circle, connected by an arrow to the laser performance for the same mouse (cyan circle). Perfectly accurate behavior corresponds to the upper left corner, and any effect of the laser toward the dashed line indicates a decrease in performance, which is seen for all mice. Most mice showed little response bias, corresponding to the direction toward the lower right corner (perpendicular to the dashed line), but two mice showed stronger laser effects on response bias (one toward the lower left corner, and one toward the upper right corner). ***G***, ***H***, Mice that did not express ChR2 showed no significant effects of laser illumination. Format as in ***E***, five mice, 2000–4000 total trials. Rewards on laser trials were either delivered randomly (***H***), or normally (***G***; i.e., for the correct response corresponding to the stimulus).

On 10% of the trials, the laser was turned on for the full 500-ms duration of the stimulus (Fig. 1*A*). Whether the laser would be turned on was decided randomly for each trial with a 10% probability. Laser trials were randomly rewarded to avoid the potential learning of new stimulus-reward associations. To control for the possibility of non-optogenetic effects of laser illumination on behavior, we also used identical fiber implantation and illumination with wild-type mice (*n* = 5 mice). Although we minimized light leakage with tight fiber connections and black paint on implants, it is conceivable that stray light from laser illumination could be distracting to the mouse, which could be a confounding effect with the intended suppression of auditory cortex. Using non-ChR2-expressing control mice isolates the effect of this potential distractor.

### Cycle suppression

To investigate the effects of prolonged suppression of auditory cortex, we used a cyclic pattern of laser activation, alternating between sets of 20 normal and 20 suppressed trials. For this cyclic laser suppression, we used 100-Hz pulsed illumination trains (5 ms on, 5 ms off) instead of stimulus-locked 500-ms laser pulses (Fig. 3*A*; Cardin et al., 2009). Output power during each 5-ms pulse was 9.5 mW. For each cycle, illumination started with stimulus request on trial 1, and ended with response selection on trial 20. Because illumination was sustained for a fixed number of trials, the actual duration of illumination depended on trial rate. In this “cycle suppression” condition, all trials were rewarded normally (according to the tone frequency), since laser trials made up 50% of the total, and we were concerned that this proportion of random rewards might disrupt overall performance.

### Sustained suppression

Sustained optogenetic suppression experiments were designed to mimic the effects of electrolytic lesions. Mice were tested for a half-hour session to provide a same-day baseline measure of performance, then received 4 h of sustained optogenetic suppression in a holding cage within the sound-attenuating behavioral chamber, using continuously pulsed 100-Hz laser illumination (5 ms on, 5 ms off, as in cycle suppression). Then, with the continuously pulsed 100-Hz laser illumination still on, mice ran for a second half-hour session. Mice remained connected to optic fibers during the entire period of sustained suppression (i.e., they were not disconnected during transfer to or from the holding cage).

### Single neuron recording and analysis

Tetrode data were acquired with 32-channel RHD2000 hardware (Intan Technologies) and Open Ephys software (Siegle et al., 2017). A minimum threshold of 60 μV was set for collection of spiking activity. Activity of individual neurons was isolated offline using MClust (Redish, 2008). Measures of peak and trough wave form voltage, energy, and principal components analysis were used as wave form separation parameters in 2-D cluster space. Cells were accepted for analysis only if they had a cluster boundary completely separate from adjacent cluster boundaries, and completely above threshold, on at least one 2-D view. Cluster boundaries were then applied across sessions to track single cell responses across different stimulus contingencies.

We recorded neuronal responses to pure tones (500-ms duration, 500-ms intertrial interval, 50 repetitions) with or without transient optogenetic suppression on interleaved trials, as well as spontaneous activity in silence before, during and after sustained optogenetic suppression (100 Hz, 100-s duration, 100-s intertrial interval, 10 repetitions). Laser power was 6.3 mW, corresponding to an irradiance of 200 mW/mm^2^ as measured at the tip of the 200-μm diameter fiber. All data were collected as mice freely explored a cylindrical plastic container (height, 16 cm; diameter, 16 cm) inside a double-walled sound-attenuating chamber. Sounds were delivered from a free-field speaker directly above the cylinder. The speaker was calibrated to within ±1 dB using a Brüel and Kjær 4939 1/4-inch microphone positioned within the cylinder approximately at head height. Following each recording session, the tetrode array was lowered ∼80 μm and allowed to settle for a minimum of 3 h before initiating another session to ensure that responses collected during each session reflected the activity of a unique population of cells. Optimal pure tone frequencies were selected by first assessing frequency tuning of individual neurons (4–64 kHz presented at 20-, 40-, 60-, and 80-dB SPL). Significantly tone-responsive cells were identified by comparing the firing rate during the first 75 ms following tone onset to an equivalent period during silence (using the paired *t* test). We determined best stimulus (frequency and intensity) from the highest firing rate response across all frequencies and intensities. Because multiple neurons were recorded simultaneously (with eight tetrodes), we selected best stimuli for several representative neurons and then presented those stimuli with and without illumination to test the effects of optogenetic suppression. After off-line spike sorting, for each cell we chose the single best frequency of those presented and tested the effects of optogenetic suppression by comparing laser-off tone responses to interleaved laser-on responses (paired *t* test, entire 500-ms tone duration). Recordings from putative PV cells, as identified by significant firing rate increases during laser pulses in silence compared with an equivalent period of silence with laser off (paired *t* test), were excluded from group analyses. To assess the effects of sustained optogenetic suppression, we compared the mean firing rate during the 100-s pulse train to that for the 10 s preceding and 10 s following the pulse train (ANOVA). We also compared the first and last 10 s of the 100-s pulse train to determine if suppression was stable over time (paired *t* test).

### Electrolytic lesions

To produce bilateral lesions of auditory cortex similar to those achieved with surgical ablations in previous studies, we passed current through implanted electrodes. For these experiments, mice were first implanted with electrodes as described above. Then, after at least 7 d of recovery, mice were trained on the task with steps 1–5 as described above (see Table 1). Mice continued running in Step 5 after performance reached asymptotic maximum, to provide several days of pre-lesion baseline data. On the day of the lesion, mice performed a half session (30 min) in the morning to establish same-day baseline, and were lesioned immediately after this session ended. Lesions used 45 s of radio frequency current (∼5 W) passed through the bipolar electrodes in each hemisphere. Mice then ran another half session in the afternoon after a median 4 h of recovery. This approach minimizes the time following the lesion (compared to the days-to-weeks recovery required after surgical ablation), providing the soonest possible testing of post-lesion performance. Mice were anesthetized with 1.5% isoflurane for ∼2 min during electrolytic lesioning. We noted that mice were disoriented for 1–2 h after lesioning, so we waited until they were alert and responsive before post-lesion testing (median 4 h). Following post-lesion behavioral data collection, we sectioned the lesioned brains and examined them to determine lesion extent. Using coronal mouse atlas sections (Paxinos and Franklin, 2019), we identified auditory cortex via landmarks such as the hippocampus and rhinal fissure and visually inspected sections spanning auditory cortex. We quantified lesion extent as the damaged fraction of total auditory cortex area across sections.

### Statistical analysis

We calculated performance separately for responses on laser and control trials. For individual mice, we measured accuracy (in percentage correct) using Fisher’s exact test on the contingency tables created by the two stimuli and two possible responses. We limited analysis to the first 10,000 total trials after the start of laser stimulation, to avoid any long-term learning effects. To examine performance as a function of time, we measured performance using a 100-trial sliding window. We tested for group effects using a one-tailed Wilcoxon signed-rank test on the control and laser performance data. We compared performance of electrolytic lesion mice before and after the lesion using the one-tailed Wilcoxon signed-rank test, comparing the performance on the final 700 pre-lesion trials to the first 700 post-lesion trials (which could span sessions). For both of these tests, the group data could be approximated as a normal distribution based on the Lilliefors’ goodness-of-fit test, so we estimated effect size using the paired Cohen’s *d*. We computed and plotted Cohen’s *d* using the tools available at https://www.estimationstats.com/, estimating the distribution with bootstrap sampling (5000 samples), and using bias-corrected and accelerated confidence intervals (Ho et al., 2019). To determine the rightward or leftward response bias of each mouse, we calculated the difference between the proportion of rightward responses and rightward stimuli, in sliding 100-trial windows. A difference of zero indicates no bias, as the choices of the mouse are proportionate to the stimuli presented. Positive values indicate that the mouse is biased to the right, and negative values indicate a bias toward the left. For display, we added 50% to the bias values, such that 50% indicates no bias, values <50% indicate leftward bias, and values >50% indicate rightward bias.

For receiver-operating-characteristic (ROC) analysis, we evaluated performance separately for each stimulus value and then compared between laser and control trials. To reveal stimulus-related effects, we separated each mouse’s performance into “13-kHz hit rate” (percentage correct on 13-kHz trials) and “13-kHz false alarm rate” (100 − percent correct on 4-kHz trials). This arbitrary assignment to hits and false alarms allows ROC analysis of the effects of laser on both performance and bias. We plotted hits against false alarms, separately for laser and control conditions for each mouse. The distance between these points indicates the magnitude of the laser effect on performance, and the direction between them indicates the degree of induced bias.

## Results

### Transient optogenetic suppression

We first tested whether transient optogenetic suppression of auditory cortex during tone presentation would impair tone discrimination. We implanted PV-ChR2 mice with optical fibers bilaterally over auditory cortex, and trained them to discriminate between two tones well-separated in frequency (4 and 13 kHz, 500 ms, ∼77 dB) to set up for optogenetic suppression during tone presentation (Fig. 1*A*).To verify that our optogenetic method effectively suppressed cortical activity, we recorded from auditory cortical neurons in separate mice (not used for behavior) using a tetrode array, attached to an optical fiber implanted in the same location as in the mice used for behavior. We recorded from 90 neurons, 46 of which responded significantly to 500-ms pure tones. We excluded PV cells, which were unambiguously identified by robust responses to illumination (Moore and Wehr, 2013). Figure 1*B* shows the effect of transient optogenetic suppression (at 200 mW/mm^2^) on the response to best-frequency tones and on spontaneous activity. Suppression was nearly complete. Across the population, suppression reduced the mean firing rate during the tone from 10.4 ± 7.2 to 1.5 ± 3.9 Hz (mean ± SD, *t* = 9.7, *p* < 0.0001, *n* = 46 tone-responsive neurons), and reduced spontaneous activity from 2.3 ± 2.4 to 0.3 ± 1.5 (*t* = 9.2, *p* < 0.0001, *n* = 90 neurons). Tone responses were significantly suppressed by illumination in 44/46 (96%) of tone-responsive neurons, and spontaneous activity was significantly suppressed in 52/90 (58%) of all recorded neurons. Note that off-responses (i.e., responses evoked by tone offset) were unaffected by suppression, because illumination ended at tone offset.

Mice achieved a high level of performance (>85%) within 2000–4000 trials (Fig. 1*C*), after which we illuminated auditory cortex just during the 500 ms tone presentation on 10% of trials (9.5 mW or 300 mW/mm^2^; Fig. 1*A*). Illumination trials were randomly rewarded to minimize the possibility that mice could learn new stimulus-response associations. Optogenetic suppression significantly, but incompletely, impaired task performance. Figure 1*D* shows an example of performance for an individual mouse over the first 10,000 trials after the onset of suppression trials. For this mouse, performance on laser trials was impaired by 10–15% compared to control trials. This was true for all mice, with performance of 90 ± 4.6% on controls trials compared to 75 ± 7.5% on laser trials (*p* = 0.0019, one-tailed Wilcoxon signed-rank test, effect size *d* = –2.35 [95.0%CI –3.15, –1.32], *n* = 9 mice; Fig. 1*E*). All mice showed significant effects (Fisher’s exact test, *p* < 0.05), with individual effects on performance ranging from 10% to 25% (Fig. 1*E*). These results show that when auditory cortex is intact and operational, mice rely at least in part on it to perform tone discrimination.

The mouse shown in Figure 1*D* did not show a strong bias on laser trials, that is, it showed roughly equal numbers of both leftward and rightward responses (Fig. 1*D*, gray line). However, some mice did develop a bias on laser trials, which could reflect a default strategy when a mouse is uncertain of the correct response. To examine the joint effects of suppression on both accuracy and bias, we turned to ROC analysis. In Figure 1*F*, the performance of each mouse on control trials is represented by an open symbol, and on laser trials by a cyan symbol. Performance on control trials is clustered at the upper left corner, indicating high accuracy and low bias. On laser trials, performance for all mice shifted toward the dashed line (chance performance). Bias is indicated by a deviation in the direction parallel to the dashed line (i.e., toward the lower-left or upper-right corners). A shift perpendicular to the dashed line (i.e., toward the lower-right corner) indicates an effect on accuracy with an absence of bias. While all mice showed significant effects on accuracy, most showed little bias, and two mice showed stronger bias (one in each direction).

We used two approaches to minimize the possibility that laser illumination could act as a visual cue that could affect behavior. First, we used black paint to minimize stray light from the optical fibers. Second, we used a color-matched continuous full-field strobe to mask any possible visual stimulation from the lasers (for example, due to intracranial retinal stimulation). To further control for the possibility that laser illumination could act as a visual cue that could affect behavior, we repeated the experiment with non-ChR2-expressing mice. We first used a “normal reward” condition, in which laser trials were rewarded normally according to the appropriate response and the only distinguishing feature of laser trials was illumination. Illumination had no effect on task performance (*p* = 0.094, one-tailed Wilcoxon signed-rank test, *n* = 5 mice; Fig. 1*G*), with individual effects on performance ranging from a 3.1% decrease to a 0.9% increase. We then used random rewards on laser trials, to exactly replicate the conditions used with ChR2-expressing mice. Illumination again had no effect on performance (*p* = 0.063, one-tailed Wilcoxon signed-rank test, *n* = 5 mice; Fig. 1*H*), with individual effects on performance ranging from a 3.5% decrease to a 0.2% increase. From this we conclude that laser illumination of the brain had no effect on task performance in mice not expressing ChR2, and thus that the effects we observed in ChR2-expressing mice were specifically due to optogenetic suppression of auditory cortex.

To address potential concerns that laser suppression could reach beyond auditory cortex, we tested a separate cohort of 6 mice on the original 300-mW/mm^2^ laser power as well as several additional sessions (1200–3200 trials) at 200 mW/mm^2^ (6.3-mW total power), which has a reduced spatial extent (Weible et al., 2014). This laser power also produced a performance deficit (*p* = 0.018, one-tailed Wilcoxon signed-rank test, *n* = 6 mice). The deficits on suppression trials were not significantly different between the two laser powers (*p* = 0.22, one-tailed Wilcoxon signed-rank test, *n* = 6 mice), suggesting that deficits can be attributed to effects on auditory cortex.

### Electrolytic lesions

**Figure 2.**
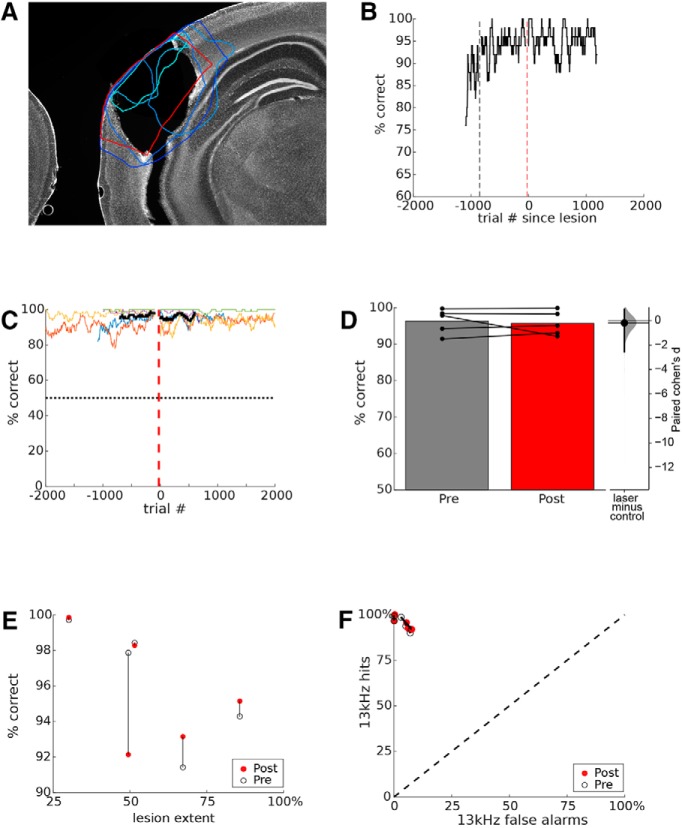
Electrolytic lesions of auditory cortex did not impair pure tone discrimination. ***A***, The section of maximal lesion extent for each mouse (*n* = 5) is shown in cyan/blue, overlaid onto the boundaries of auditory cortex (red). ***B***, Performance before and after lesion for an example mouse (mouse 6685). Vertical black dashed line indicates graduation to the final training step (6). In the 700 pre-lesion trials, overall performance was 94.25% correct. The lesion is indicated by the red dashed line at trial zero. In the 700 trials following the lesion, the mouse performed at 95.1%, showing a lack of impairment. Performance is computed in a 100-trial sliding window. Chance performance is 50%. ***C***, Performance for each of five mice is shown in different colors. Format as in ***B***. Traces are aligned to the time of the lesion, indicated by the red dashed line. Note that a median of 4 h elapsed at the time of lesion (red dashed line). Heavy black line shows mean across mice for 700 trials before and after lesion. ***D***, Mean performance across all mice on the 700 trials pre-lesion and 700 trials post-lesion are represented as bars, with the performance of individual mice indicated by connected dots. Inset at right shows estimated effect size, format as in Figure 1*E*. ***E***, Lesion extent for individual mice is plotted against their pre-lesion performance (black) and post-lesion performance (red). ***F***, ROC analysis (same format as Fig. 1*F*). Open circles indicate pre-lesion performance for each mouse, which are connected to red circles indicating post-lesion performance. Lesions had negligible effects on accuracy or response bias.

The fact that transient optogenetic suppression of auditory cortex impaired tone discrimination performance is surprising, because permanent lesions of auditory cortex have no effect on pure tone discrimination (Butler et al., 1957; Goldberg and Neff, 1961; Ohl et al., 1999). Typical lesion studies require a recovery period of days or longer after surgery, and we wondered whether long-term plasticity or reorganization during this recovery period could contribute to the marked difference between the effects of lesions and transient optogenetic suppression. To address this, we implanted electrodes for producing electrolytic lesions of auditory cortex, and trained mice on tone discrimination after recovery. This allowed us to lesion auditory cortex bilaterally and then test the effects on tone discrimination within a few hours. Lesions were extensive, averaging 56.8 ± 9.3% of AC and 69.3 ± 10.0% of A1 (Fig. 2*A*). After mice reached stable asymptotic pre-lesion performance, we ran them for a half-hour session in the morning, delivered electrolytic lesions, and ran them again the same day after recovery (median recovery time 4 h). Mice continued to run the task on subsequent days. Lesions had no effect on tone discrimination performance, either in individual mice or at the group level (Fig. 2*B–D*). Mean pre-lesion performance was 96.3 ± 1.5% and post-lesion performance was 95.7 ± 1.5% (*p* = 0.45, one-tailed Wilcoxon signed-rank test, effect size *d* = –0.19 [95.0%CI –2.55, 0.938], *n* = 5 mice; Fig 2*D*). Since there was variation in lesion extent, we tested whether this was correlated with behavioral effects (Fig. 2*E*) and found that it was not (Spearman’s correlation, *r* = 0.6, *p* = 0.35). ROC analysis showed no effect of lesions on accuracy or bias (Fig. 2*F*). These results show that when auditory cortex has been extensively damaged, within 4 h, mice are able to use alternative circuits to perform tone discrimination with no measurable deficit.

In some tasks, overtraining has been shown to shift task dependence from cortex to striatum as performance becomes habitual (Smith and Graybiel, 2013). We therefore wondered whether different amounts of training time could have contributed to the differences we observed between the effects of optogenetic suppression and lesions. However, there was no difference between groups in the number of training trials before the manipulation, either for all training (steps 1–5, *p* = 0.89, rank-sum) or for the final stage of training (step 5, *p* = 0.61). Thus, groups had no systematic differences in overtraining that could explain the different effects of optogenetic suppression and lesions.

### Cycle suppression

**Figure 3.**
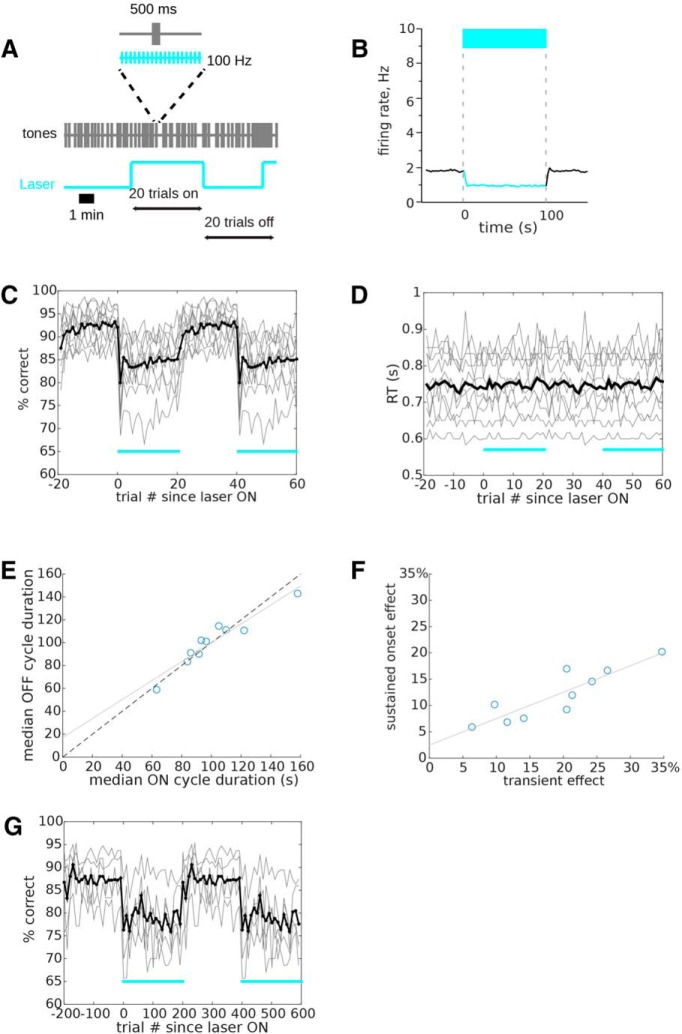
Cycle suppression. ***A***, We designed a cycle suppression protocol in which a 100-Hz laser pulse train (300 mW/mm^2^, 5 ms on/off or 50% duty cycle) was on for 20 consecutive trials, and then off for 20 consecutive trials, in alternating blocks. Blocks of 20 trials had median durations of 1–3 min. ***B***, Electrophysiological validation of optogenetic suppression using a 100-Hz laser pulse train in separate mice. We used a fixed duration of 100 s (corresponding to the approximate duration of 20 trials) and measured the effect on spontaneous activity in silence (10 repetitions, *n* = 153 neurons). The suppression of spontaneous activity was significant and stable throughout the duration of the pulse train. ***C***, Performance on each trial within the cycle, averaged across a minimum of 119 blocks, is shown for individual mice in gray, with the mean across all mice in black (*n* = 10 mice). Cyan bars indicate the trial blocks with the laser on. The data are duplicated to display two complete cycles. Mice showed a sharp initial drop in performance on the first trial in an on-cycle, followed by a rapid but partial recovery. Performance did not return to baseline performance by the end of the 20-trial block. ***D***, Median reaction time on each trial within the cycle, averaged across all cycles, is shown for individual mice in gray, with the median across all mice in black. Reaction times were unaffected by laser. ***E***, Median 20-trial cycle duration for the on and off cycles of each mouse. Median on-cycle duration and off-cycle duration were tightly correlated and nearly equal, indicating that mice showed individual differences in trial rate but no difference between response rates during on-cycles and off-cycles. Gray line is a linear regression, dashed line is unity. ***F***, After testing on 20-trial cycles, we re-tested all 10 mice with transient 500-ms suppression (as in Fig. 1*A*). We compared the effect size for cycle suppression to the effect size for transient 500-ms suppression, for each mouse. We quantified effect size for cycle suppression as the drop in performance from the last trial in the off-cycle to the first trial in an on-cycle. Effect size was tightly correlated between the two suppression protocols, indicating that mice showed stable individual differences in the effects of suppression. The effect size for transient suppression was roughly double the effect size for cycle onset. Gray line is a linear regression. ***G***, To test cycle suppression over a longer time course, we extended the cycle protocol shown in A to span 200 trials with the laser pulse train on, alternating with 200 trials with laser off. Performance on each trial within the cycle is shown in same format as ***B***, except that data are binned (in 10-trial bins). Mice did not show a full recovery to control performance even after 200 trials (median duration 20.5 min).

One interpretation of the lesion and transient suppression experiments described above is that together they provide an upper and lower bound on the time scale required for mice to recover the ability to discriminate tones when auditory cortex becomes unavailable. That is, transient (500 ms) unavailability of auditory cortex produces a deficit, but within ∼4 h, some recovery process allows accurate performance even without auditory cortex. The time course for this recovery process must then lie between 500 ms and ∼4 h. To better understand this putative recovery process, we set out to characterize its time course. Because any given trial response is either correct or incorrect, estimating performance accuracy requires integration across trials. For example, at least 100 trials are required to estimate performance (in percentage correct) with a precision of 1%. Because our mice perform approximately five trials per minute, 100 trials took 20 min on average. This means that the temporal precision achieved by integrating consecutive trials is insufficient to resolve the fine time scale of the recovery process. We therefore designed a cyclical suppression paradigm so that we could average performance over large numbers of trials with high temporal precision (Fig. 3*A*). To verify that cycle suppression effectively suppressed cortical activity, we tested this method on a population of 153 auditory cortical neurons recorded with tetrodes (including the cells shown in Fig. 1*H*, and excluding PV cells). Because tetrode-implanted mice did not perform the task, instead of 20 trials of continuous suppression, we used a fixed duration of 100 s (corresponding to the approximate duration of 20 trials; see Fig. 3*E*). Across the population, suppression reduced spontaneous activity from 1.8 ± 1.9 to 0.9 ± 1.4 Hz (*t* = 9.2, *p* < 0.0001, *n* = 153 neurons; Fig. 3*B*). Spontaneous activity was significantly suppressed in 102/153 (67%) of neurons. Suppression was stable throughout the 100-s suppression period, with no mean difference between the first 10 s of suppression and the final 10 s (*p* = 0.60).

After a new group of 10 fiber-implanted PV-ChR2 mice reached asymptotic performance on step 5, we alternated 20-trial blocks of no illumination with 20-trial blocks of 100-Hz illumination. Figure 3*C* shows the performance of individual mice as well as the group mean averaged across a minimum of 119 blocks, representing at least 19 d of behavior. On trial 1 (the first illumination trial in the cycle), laser onset coincided with tone onset, making it analogous to the laser trials in the 500 ms transient suppression experiment, producing a 12% drop in performance (*p* = 0.00098, one-tailed Wilcoxon signed-rank test). Already by the second trial in the cycle, performance recovered substantially. However, performance did not recover to pre-suppression levels by the end of the cycle (comparison of last suppression trial with last pre-suppression trial: *p* = 0.002, one-tailed Wilcoxon signed-rank test). Reaction time was unaffected by optogenetic suppression (Fig. 3*D*) regardless of position within the trial cycle (comparison of first suppression trial with last pre-suppression trial, *p* = 0.422, one-tailed Wilcoxon signed-rank test). Mice completed the 20-trial cycles in ∼1–3 min, which did not differ between laser-on and laser-off blocks (*p* = 0.461, one-tailed Wilcoxon signed-rank test; Fig. 3*E*). This indicates that prolonged suppression produces an immediate deficit followed by a rapid but partial recovery, as well as a sustained deficit lasting at least 20 trials or ∼1–3 min. Recovery was not well fit by either single or double exponentials, either with respect to trials or to elapsed time, suggesting a complex time course.

After mice completed at least 19 d of testing with the 20-trial cycle protocol, we tested them with transient 500-ms suppression to compare the strength of effects. Mice showed individual differences in effect sizes, but across mice the effect of transient 500-ms suppression was highly correlated with the effect of cycle suppression (*R* ^2^ = 0.7834, *p* = 0.0007, linear regression, *n* = 10 mice; Fig. 3*F*). The effect size for the initial trial of cycle suppression was half of that for transient 500-ms suppression (regression slope m = 0.502, y-intercept = 2.5%). This could be due to the 50% duty cycle of the sustained 100-Hz pulse train (5 ms on, 5 ms off).

We wondered whether the incomplete recovery seen after 20 trials of suppression (Fig. 3*C*) indicated the existence of a longer-lasting recovery process. We therefore extended this cycle-based approach to use cycles of 200 trials with laser on, alternating with 200 trials with laser off. The initial laser onset deficit remained, as seen from a comparison of performance on the final 10 pre-suppression and first 10 post-suppression trials (*p* = 0.031, one-tailed Wilcoxon signed-rank test). Mice showed a sustained performance deficit even after 200 trials (*n* = 5 mice; Fig. 3*G*). The deficit in the last 10 trials was no different from that in the first 10 trials (*p* = 0.31, one-tailed Wilcoxon signed rank test), nor was it different from the last 10 trials using the 20-trial cycle protocol (*p* = 0.5, one-tailed Wilcoxon signed rank test). There was also no trend toward recovery over the course of the 200 trials (linear regression slope m = –0.032, *p* = 0.686). Mice completed the 200-trial cycles in a median (across mice) of 20.5 ± 5.3 min. This suggests that if there is a recovery process during optogenetic suppression of auditory cortex, it lasts at least 20.5 min. Given the lack of any trend toward recovery (Fig. 3*G*), it seems likely that complete recovery would take appreciably longer than 20.5 min.

Given that we observed a complete recovery following electrolytic lesions of auditory cortex, tested 4 h after lesion, these results suggest that either (1) recovery after cortical lesion/suppression has a time course that lies between 20.5 min and 4 h, or else (2) electrolytic lesions and optogenetic suppression have fundamentally different effects on tone discrimination performance. To distinguish between these possibilities, we designed a sustained optogenetic protocol to mimic as closely as possible the time course of our electrolytic lesion experiments.

### Sustained suppression

**Figure 4.**
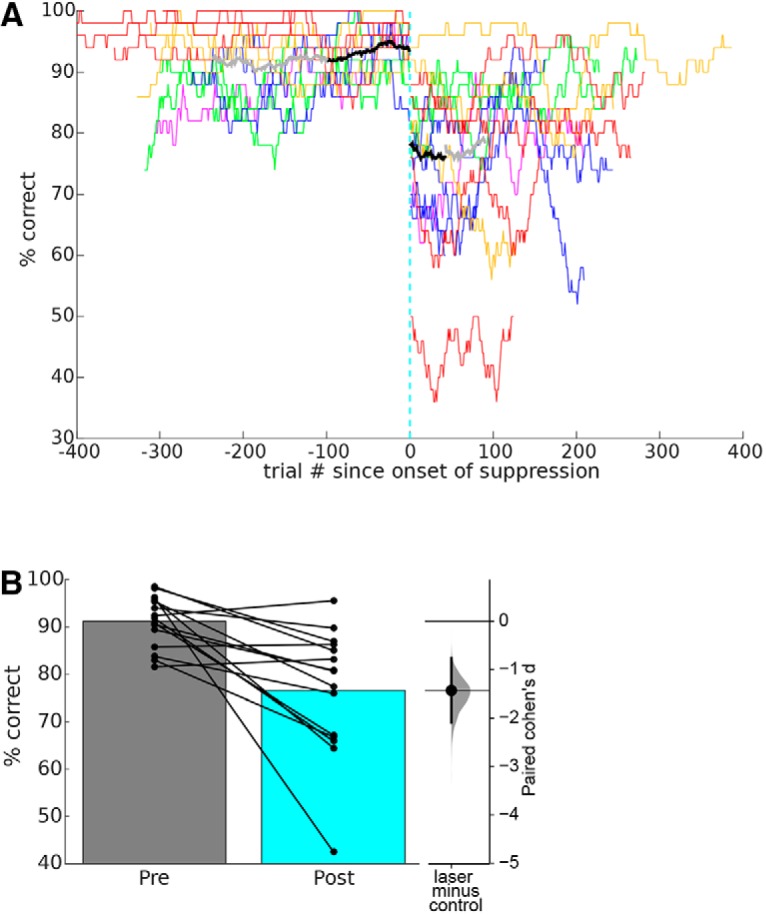
Sustained optogenetic suppression impairs pure tone discrimination. ***A***, Performance for each of five mice before and after 4 h of sustained optogenetic suppression (format as in Fig. 2*C*; each mouse is shown in a different color). Note that 4 h elapsed during sustained suppression (cyan dashed line), after which suppression was maintained throughout a second session of behavior. We repeated this experiment one to four times per mouse, resulting in 15 sessions for the five mice. Because mice ran a different number of trials in each session, we averaged across mice by including only trials contained in all 15 sessions (black line, which is limited to the duration of the shortest session). The gray line shows the average across the 14 sessions not including the shortest one. ***B***, Mean performance before and after sustained suppression. Format as in Figure 2*D*. Connected dots show each mean performance for each of 15 sessions (using trials from the black segments in ***A***). Inset at right shows estimated effect size, format as in Figure 1*E*.

In our electrolytic lesion experiments, we tested well-trained mice for half-hour in the morning to establish same-day baseline performance, then electrolytically lesioned auditory cortex bilaterally (45 s of current), and re-tested mice as soon as they were bright, alert, and responsive (median 4 h post-lesion). To optogenetically mimic this protocol, we replaced the electrolytic lesion step with an onset of sustained 100-Hz laser illumination. We ran mice on tone discrimination for a half-hour, then connected fibers and started a sustained 100-Hz laser pulse train, and allowed mice to remain in a holding cage in the testing chamber for 4 h of sustained 100-Hz illumination. Then, with continued laser illumination, we re-tested tone discrimination for at least 30 min. Mice showed a sustained performance deficit after 4 h of sustained illumination (Fig. 4). Mean pre-suppression performance was 93.4 ± 1.4%, and after 4 h of suppression was 76.6 ± 3.4% (*p* = 0.0012, one-tailed Wilcoxon signed-rank test, effect size *d* = –1.43 [95.0%CI –2.09, –0.764], *n* = 15 sessions in five mice). This indicates that the tone discrimination deficit produced by sustained optogenetic suppression persists for at least 4 h. From this we conclude that electrolytic lesions and optogenetic suppression have fundamentally different effects on tone discrimination performance: lesions of auditory cortex have no effect on performance as soon as it can be measured, but suppression over the same time course produces a lasting deficit.

## Discussion

Here, we used both optogenetic suppression and lesions to ask whether auditory cortex is required for frequency discrimination of pure tones in mice. We found that lesions of auditory cortex had no effect on tone discrimination, even when tested only 4 h after the lesion. However, optogenetic suppression of auditory cortex significantly impaired tone discrimination, across a wide range of durations of suppression. Transient suppression just during tone presentation produced the strongest deficit. Performance during sustained suppression recovered rapidly after the first trial of suppression, but only partially, quickly stabilizing within a few trials to a persistent deficit. This deficit remained for as long as suppression was maintained, up to at least 4 h. These results suggest that lesions and optogenetic suppression of auditory cortex produce fundamentally different effects on tone discrimination performance, which cannot be explained simply by the time course of some recovery process. Rather, it seems likely that multiple redundant systems must contribute to tone discrimination. This suggests that the absence of auditory cortex induces switching between these systems, but that the efficacy and time course of this switching process depends strongly on the method by which auditory cortex is made unavailable. We conclude that auditory cortex does contribute to frequency discrimination for pure tones. This is surprising given that we and others have shown that lesions have no effect on tone discrimination.

Numerous lesion studies over the past 75 years have investigated the role of auditory cortex in frequency discrimination (Meyer and Woolsey, 1952; Butler et al., 1957; Thompson, 1960; Goldberg and Neff, 1961; Kelly, 1970; Sellick, 1983; Buser and Imbert, 1992; Ohl et al., 1999; Talwar et al., 2001; Rybalko et al., 2006; Porter et al., 2011; Gimenez et al., 2015). Although there are conflicting results, the consensus view is that frequency discrimination is not affected by lesions of auditory cortex, even extensive lesions of all known auditory cortical fields. Cortical lesions have been shown to produce deficits if the stimuli are more complex (such as frequency-modulated tones, or complex tones with a missing fundamental frequency), or if a temporal judgment is required, or with greater task difficulty or more elaborate testing or training procedures. For pure tones, however, it appears that some other brain region or system can support frequency discrimination when auditory cortex is destroyed. In contrast, transient inactivation of rat auditory cortex with muscimol has been reported to completely eliminate tone detection (as if animals were totally deaf), with coarse frequency discrimination recovering over several hours as muscimol gradually wears off, followed by recovery of fine frequency discrimination (Talwar et al., 2001). It has been difficult to reconcile these strikingly different effects of lesions and muscimol. One possible explanation is that muscimol, a small molecule, could diffuse to and inactivate other cortical or subcortical auditory structures. Yet auditory cortical lesions have been shown to produce extensive degradation of the MGN (Kelly, 1970), without impacting tone discrimination, suggesting that muscimol must diffuse at least to inferior colliculus or brainstem structures in order for diffusion to account for the difference from lesion results. Other studies have reported that muscimol inactivation of auditory cortex had only a slight (but significant) impact on frequency discrimination (effect size ∼6%; Gimenez et al., 2015). An important difference between these studies is that Talwar et al., used a 20-fold higher dosage of muscimol, which could support greater diffusion (but not a difference in the degree of inactivation of auditory cortex, which was nearly complete in both studies). Other differences such as task design and drug administration methods could also play a role. A different possible explanation for the discrepancy between lesion and some muscimol inactivation studies is that the recovery process, or switching of task implementation from auditory cortex to some other brain structure (such as inferior colliculus), is engaged by injury but not by inactivation. Our results support this interpretation. Our findings that optogenetic suppression impaired tone discrimination, but lesions did not, even when the time course was similar for both (4 h), are consistent with the idea that injury induces a rapid recovery process that inactivation alone does not. The fact that the time course, task design, behavioral apparatus, and species were identical for both our lesion and optogenetic suppression experiments greatly constrains the possible factors that could lead to recovery or the lack thereof.

Our results indicate a role for auditory cortex in tone discrimination, but it remains unclear what the nature of this role is. For example, optogenetic suppression of auditory cortex could produce a deficit in sensory processing, or could instead interfere with the integration of the sensory stimulus and the appropriate motor response (Kuchibhotla and Bathellier, 2018). For example, it is possible that both auditory cortex and an alternative circuit normally operate to discriminate frequencies, but the alternative circuit is ignored for the purposes of decision-making as long as there is some auditory cortical activity on which to base a choice. At the sensory processing level, there are conflicting reports about whether lesions of auditory cortex affect hearing thresholds (Butler et al., 1957; Goldberg and Neff, 1961; Neff et al., 1975; Heffner and Heffner, 1986, 1990). We designed our stimuli to be easily discriminable, with a sound level of 77 dB and a wide >1.5 octave frequency separation. This suggests that a change in hearing thresholds would need to be substantial to account for our results. The fact that suppression had no effect on response times (Fig. 3*D*,*E*) helps to rule out effects on motivation or arousal, or effects due to distraction or motor disruption.

A role for auditory cortex in frequency discrimination has also been shown by modulation of cortical activity, rather than complete suppression. Interestingly, moderate photoactivation of PV interneurons in auditory cortex, using a PV-ChR2 strategy similar to ours, has been shown to improve frequency discrimination acuity (Aizenberg et al., 2015). In that study, PV activation using a low laser power (0.2 mW/mm^2^) had a stronger effect on spontaneous than on tone-evoked activity, leading to a net increase in tone-evoked signal-to-noise ratio. This suggests that enhanced population coding in auditory cortex underlies the improvement in frequency discrimination thresholds seen with low-power photomodulation (Aizenberg et al., 2015; Briguglio et al., 2018). In contrast, we used a thousand-fold greater laser power (300 mW/mm^2^), which abolished tone responses altogether rather than enhancing signal-to-noise.

One mechanism that has been proposed to explain differing effects of lesions and optogenetic suppression is the loss of a biasing input to downstream structures (Otchy et al., 2015; Hong et al., 2018). In this scenario, loss of activity in auditory cortex would cause a loss of synaptic drive to downstream neurons, acutely dropping their membrane potentials below firing threshold and impairing the flow of information through circuits supporting discrimination performance. Due to homeostatic mechanisms such as an increase in membrane excitability, these downstream neurons respond to a sustained lack of firing by lowering spike thresholds, allowing a previously sub-threshold alternative synaptic pathway to drive spiking output. Homeostatic synaptic plasticity or shifts in the balance of excitation to inhibition could achieve similar results (Scholl and Wehr, 2008). Thus, acute optogenetic suppression could impair circuit function, but on a longer homeostatic time scale, alternative circuits could support recovery from a lesion. Key to this model is the difference in time scale between acute optogenetic suppression and longer-term recovery from a lesion. Our results appear to rule out this scenario, because in our sustained suppression experiment, we maintained optogenetic suppression for as long as animals took to successfully recover from a lesion and perform without deficit. This indicates that any homeostatic mechanism arising from a loss of cortical activity should have had enough time to permit recovery through an alternative pathway.

Similar to our findings, optogenetic suppression of somatosensory cortex partially impairs a whisker-based object detection task (Hong et al., 2018). However, somatosensory cortical lesions also produced a partial impairment on that task, in contrast to our findings. Interestingly, object detection performance recovered abruptly to pre-lesion levels by the next session, and task exposure appeared to be instrumental for this recovery. We found that mice showed no deficit after lesions, even within the first few trials (Fig. 2*B*,*C*), suggesting that task exposure is not necessary for recovery under our experimental conditions. Dorsolateral striatum appeared to be necessary for recovery after somatosensory cortical lesions (Hong et al., 2018), raising the possibility that the striatum might also be involved in the rapid recovery of tone discrimination ability after lesions to auditory cortex.

## Synthesis

Reviewing Editor: Darcy Kelley, Columbia University Decisions are customarily a result of the Reviewing Editor and the peer reviewers coming together and discussing their recommendations until a consensus is reached. When revisions are invited, a fact-based synthesis statement explaining their decision and outlining what is needed to prepare a revision will be listed below. The following reviewer(s) agreed to reveal their identity: NONE. Note: If this manuscript was transferred from JNeurosci and a decision was made to accept the manuscript without peer review, a brief statement to this effect will instead be what is listed below.

Please submit a revised version with the estimation statistics. Also: mice for miced on line 134.
